# Psychological Risk and Resilience of Older People’s Exposure to Climate Change

**DOI:** 10.1093/geroni/igab046.2259

**Published:** 2021-12-17

**Authors:** Toni Antonucci

**Affiliations:** University of Michigan, Ann Arbor, Michigan, United States

## Abstract

Climate change places older people at physical and psychological risk. Even small changes in temperature (+/- 1 degree) results in increased morbidity and mortality. Further, the burden of climate change is not borne equally. The stress and mental ill health associated with disasters are especially borne by women, older adults, persons of color, low-income populations, those with preexisting conditions, poor support networks, and those residing in sub-standard housing. Older people are disproportionately represented in these groups, but interventions can ameliorate these risks. At the same time, older people, with the wisdom of experience, can be important assets for families and communities struggling with the effects of climate change. Both addressing the vulnerabilities and enhancing the strengths of older people can serve to meet the immediate needs and the long-term SDGs goals of (3) health and well-being (5) gender equality (10) reduced inequalities (11) sustainable cities and communities (13) climate action.

